# Psychological model of hierarchical classification for body regulation practices

**DOI:** 10.1192/j.eurpsy.2022.1528

**Published:** 2022-09-01

**Authors:** E. Nikolaev, S. Petunova

**Affiliations:** Ulianov Chuvash State University, Social And Clinical Psychology Department, Cheboksary, Russian Federation

**Keywords:** body regulation practices, hierarchical model, Body image dissatisfaction

## Abstract

**Introduction:**

Body image dissatisfaction entails an activity, which is nothing else but an attempt for deliberate regulation of their body. It has different kinds and manifestations. Most researchers focus on such body regulation practices as weight control, muscles build-up, or cosmetic surgery.

**Objectives:**

Our goal is to work out a psychological hierarchical model of body regulation practices aimed at abating a person’s dissatisfaction with their body image.

**Methods:**

Using a method of agglomerative hierarchical clustering, we carried out a multivariate classification of 122 respondents’ answers to the Body Regulation Practices Survey (E. Nikolaev), which allows establishing the frequency of the respondents’ use of each of the 11 variants of body regulation practices offered in the survey.

**Results:**

Based on the results of 11 variables of a dendrogram, we established two data arrays, combining correspondingly 4 and 7 versions of body regulation practices. The first array comprises two pairs of clusters – physiological practices and weight control, as well as practices of personality and spiritual development. We identified it as “developmental body regulation practices”. The second array includes two paired clusters – aesthetic medicine and body modifications; image making and hetero-aggressive practices. Merging with the four practices mentioned above on a higher level of the hierarchy are auto-aggressive and inertial practices. We identified this array as “compensatory – non-adaptive body regulation practices”.

**Conclusions:**

The devised model can become the basis for further advanced research in the area of body regulation in cases of dissatisfaction with body image.

**Disclosure:**

No significant relationships.

